# Cognitive enhancement by drugs in health and disease

**DOI:** 10.1016/j.tics.2010.11.002

**Published:** 2011-01

**Authors:** Masud Husain, Mitul A. Mehta

**Affiliations:** 1 UCL Institute of Cognitive Neuroscience and UCL Institute of Neurology, 17 Queen Square, London WC1N 3AR, UK; 2 Department of Neuroimaging, Centre for Neuroimaging Sciences (PO89), Institute of Psychiatry, King's College London, London SE5 8AF, UK

## Abstract

Attempts to improve cognitive function in patients with brain disorders have become the focus of intensive research efforts. A recent emerging trend is the use of so-called cognitive enhancers by healthy individuals. Here, we consider some of the effects – positive and negative – that current drugs have in neurological conditions and healthy people. We conclude that, to date, experimental and clinical studies have demonstrated relatively modest overall effects, most probably because of substantial variability in response both across and within individuals. We discuss biological factors that might account for such variability and highlight the need to improve testing methods and to extend our understanding of how drugs modulate specific cognitive processes at the systems or network level.

## Uses of cognitive enhancement

In the last decade, pharmacological treatments aimed at improving cognitive function across a range of brain disorders have been explored and have even become established in clinical practice [1]. In developmental conditions such as attention deficit hyperactivity disorder (ADHD), drugs acting on the noradrenergic and dopaminergic systems, such as methylphenidate and atomoxetine, are now in widespread use [2–4]. For neurodegenerative disorders such as Alzheimer's disease and Parkinson's disease, acetylcholinesterase inhibitors (AChEIs) and memantine [an *N*-methyl-D-aspartate (NMDA) receptor antagonist] are now standard treatments [5–9].

In chronic mental disorders such as schizophrenia, cognitive deficits are a separable feature from positive (e.g. hallucinations and delusions) and negative (e.g. blunted affect, poverty of speech) symptoms, with current antipsychotic treatments having little, if any, impact on cognitive impairments. A wide range of compounds is therefore being assessed for cognitive enhancement in this disorder [10]. Similarly, attempts to ameliorate cognitive deficits following stroke are being actively explored [1,11–13], although none have been established. Many such cognitive enhancers target neuromodulatory systems – cholinergic, dopaminergic, noradrenergic and serotonergic – ascending from brainstem nuclei to innervate both cortical and subcortical systems (Table 1).

Although most of the reported positive effects of such drugs have been modest in magnitude overall and are highly variable across individuals, they have had an enormous impact, stimulating interest in cognitive enhancement not only for patients with brain disorders, but also for healthy individuals. Compounds such as methylphenidate and modafinil are used by students in pursuit of better grades, military personnel who need to remain awake for long missions, elderly individuals afraid of cognitive decline and even university academics keen to maintain their performance [14–17].

Here we focus on what aspects of cognition are enhanced, the magnitude of these effects and possible mechanisms underlying variations in response across individuals. Our aim is to highlight key common themes across studies of clinical populations and healthy individuals, using examples that highlight these principles. Other recent reviews provide excellent discussions of ethical issues in cognitive enhancement [18] and illustrate the complexity of physiological, cellular and computational mechanisms underlying such effects [19–22].

## What is enhanced?

What exactly do cognitive neuromodulators do? It might be tempting to assume a selective one-to-one mapping between a specific neurotransmitter system and a particular cognitive function. For example, dopamine has been strongly linked with working memory (WM) and attention [19], whereas serotonergic drugs have been prominently associated with affective processes [23,24]. However, serotonergic modulation can also influence WM [25], as can noradrenaline and acetylcholine. Conversely, dopamine influences affective processing [26,27]. A simple mapping between a specific neurotransmitter and a particular cognitive function described at a very general level – such as WM – therefore seems untenable. However, subtle but important differences in the precise processes modulated might provide some discriminating value: for instance, dopamine has an established role in reinforcement learning in response to rewards [28,29], whereas serotonin seems to modulate reinforcement learning for aversive stimuli [20,23].

To add to the complexity, neurotransmitters act via a suite of different receptor systems. Thus, dopamine acting at D1 receptors can have very different – even opposing – effects to that of its actions at D2 receptors [19,30]; for serotonin there are 17 different receptor systems. In addition, dopamine can have very different effects at different brain regions, even within different regions of the human basal ganglia [31]. Its release can also be modulated in a highly specific regional manner by other neurotransmitters, such as glutamate within the nucleus accumbens [32]. Thus, interactions between neuromodulatory systems are also a probable mechanism by which some of their effects are modulated. For instance, dopamine, noradrenaline and acetylcholine release is under histaminergic H3 heteroreceptor control [33], whereas noradrenaline and dopamine can interact to modulate spatial WM neuronal responses in prefrontal cortex in a synergistic fashion [19,21]. Again, these considerations suggest that simple conceptualizations linking a specific neurotransmitter to a single cognitive function are unlikely to be helpful.

Finally, there is increasing evidence that several neurotransmitters might have different modes of action when released in a tonic, sustained manner compared to phasic release [29,34,35]. For instance, baseline firing of noradrenergic cells in the locus coeruleus varies with different states of alertness or arousal. Optimal responses to environmentally important events seem to be linked to phasic firing of these cells, but this occurs only when tonic levels of activity are moderate [35]. Thus, alteration of global concentrations of a neurotransmitter might modulate the ability to respond to external events mediated by phasic firing.

How do drugs currently used as enhancers produce their beneficial effects? Is it through multiple effects on several different cognitive processes or do they enhance one cognitive mechanism – such as arousal or improved sustained attention – through which they lead to better performance across a battery of tests? For studies in clinical populations, the difficulty is that many standard cognitive test batteries used in clinical trials are very unlikely to be sensitive enough to answer questions on the specificity of cognitive modulation (Box 1).

For example, AChEIs such as rivastigmine and donepezil are now widely used to treat Parkinson's disease dementia (PDD) and the related condition of dementia with Lewy bodies (DLB). Many clinical trials have reported modest global beneficial effects of such drugs on bedside cognitive screening tests [5–7]. More detailed assessment using sensitive computerized cognitive tests has revealed widespread improvements in the domains of attention, WM and episodic memory [36–38]. However, these positive effects of AChEIs might all be mediated via a common process such as elevated arousal [39,40]. In fact, the very same issue pertains to the modulatory effects of AChEIs in healthy subjects [41]. For example, in young volunteers, donepezil improves episodic memory, whereas healthy elderly subjects show improvements in verbal memory [42]. Is it possible that these effects could be due simply to a generalized improvement in arousal? Studies demonstrating that donepezil attenuates decline in short-term memory and visual attention induced by sleep deprivation [43,44] raise the possibility that this might indeed be the case.

Similar considerations as for AChEIs also apply to modafinil, which has become a popular drug for cognitive enhancement in healthy individuals. Although its precise mechanism of action remains to be established, modafinil is used as a wake-promoting agent for the treatment of narcolepsy, a disorder associated with excessive daytime somnolence. Analysis of the effects of modafinil in healthy subjects has revealed improvements in attention, memory and executive function in sleep-deprived individuals [17]. However, this might simply be due to improved wakefulness or arousal induced by the drug [17], just as caffeine can improve performance on a variety of measures, including vigilance, and on incidental learning and WM tests [45]. However, it is also important to appreciate that ‘arousal’ need not be a unitary process: there is evidence of different arousal systems that might be selectively modulated by different types of pharmacological intervention [46].

**Figure 1:**
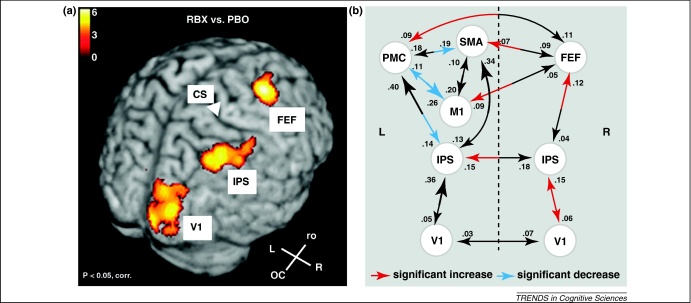
Network effects of reboxetine in visuomotor control. (**a**) The noradrenaline reuptake inhibitor reboxetine improved visuomotor control in healthy volunteers and increased cortical activity in the right intraparietal sulcus (IPS), frontal eye field (FEF) and primary visual cortex (V1). (**b**) Dynamic causal modelling demonstrated enhanced coupling between these regions when participants were on reboxetine (adapted from with permission from [50]).

It is possible that neuroimging studies might contribute to identification of the mechanisms underpinning improvement on cognitive tests, including arousal. Although early studies assessed changes in brain activity on drug administration [47–49], more recent investigations have begun to examine the modulatory effect of compounds on brain networks. For example, the beneficial effects of reboxetine on visuomotor control are associated with strengthening of coupling between selective regions in posterior and anterior regions of the right hemisphere (Figure 1) [50]. Approaches to characterize the effects of drugs at a network level in brain disorders are also being applied in patient groups (Box 2). Finally, it is also crucial to appreciate that non-cognitive factors such as alterations in mood, anxiety, motivation or apathy induced by a drug can have indirect effects on cognition. Hence, it is useful to control for these factors if at all possible.

## How effective are the benefits?

A major issue in assessing cognitive enhancement studies is the problem of effect size. First, in studies of healthy subjects, there is no universal, standard battery of tests that has been agreed on, so comparisons across studies are not easy. It is not possible to compare effect sizes for different drugs if the tests used differ in the level of difficulty or method of measurement (e.g. reaction time vs error rate). Overall, however, the effects of cognitive enhancers such as methylphenidate, modafinil and AChEIs in healthy individuals seems to be quite modest according to recent systematic reviews [17,41]. Second, many experimental investigations in healthy subjects have used single-dose assessments aimed primarily at assessing mechanisms rather than establishing optimal cognitive enhancement. Very few studies have examined the effects of repeated doses or long-term effects, which might be far more revealing and representative of the overall costs and benefits of taking cognitive enhancers on a regular basis. Third, as we have seen, although clinical trials in patients often use standardized bedside batteries, they might be hampered by their insensitivity and limited range of measurement (Box 1).

Nevertheless, even for these relatively crude measures, studies in clinical populations have revealed significant effects of long-term drug use that have led to changes in practice. For example, one of the remarkable changes in the management of neurological conditions in the last decade has been the advent of treatment for cognitive deficits in neurodegenerative conditions, initially in Alzheimer's disease with AChEIs [7]. These studies stimulated clinical trials in other conditions such as PDD and DLB, with two major placebo-controlled studies involving over 650 patients demonstrating significant positive effects of the AChEI rivastigmine on cognition and neuropsychiatric measures such as apathy, anxiety and visual hallucinations [5,6].

**Figure 2:**
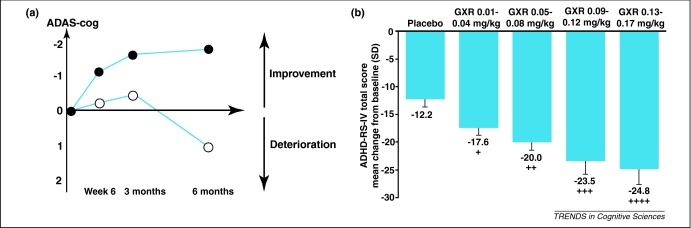
Effect sizes of cognitive enhancers in clinical studies. (**a**) Overall change in ADAS-Cog scores over 6 months on an AChE inhibitor in probable vascular dementia and Alzheimer disease patients (black circles) compared to patients on placebo (white circles) (adapted from with permission from [89]). (**b**) Improvements in ADHD Rating Scale IV with guanfacine at different doses versus placebo over 9 weeks in children and adolescents with ADHD (adapted from with permission from [90]).

Although these trials have now led to widespread clinical use of rivastigmine, it is important to keep the effect size in perspective. In the larger study, rivastigmine produced only a mean 2-point improvement on the ADAS-Cog battery (Box 1), which has a 70-point range [6]. Similar degrees of change have been observed in Alzheimer's disease and vascular dementia trials with AChEIs (Figure 2a).

Of course, effect sizes vary across individual patients. Indeed ∼40–80% of PDD or DLB patients might not show a response to treatment on such clinical measures, but other individuals show a very strong improvement [5,6]. Overall, therefore, this means that positive effects have been moderate, at best, when results are examined at the group level – at least using this currently accepted method for measuring cognition in neurodegenerative clinical trials. Similar conclusions have been reached in schizophrenia, for which there is currently no established treatment for cognitive enhancement [10]. Thus, interindividual variability might be one potential reason for small overall effect sizes (see below).

By contrast, a first glance might indicate far more substantial effect sizes in treatment trials of ADHD, for which several drugs that target the catecholaminergic system are used in clinics. For example, a recent study using high levels of the α2 noradrenergic agonist guanfacine demonstrated a 12-point mean improvement compared to placebo on a rating scale with a range of 54 points (Figure 2b). However, these effects were based on ratings by parents or caregivers, and not on cognitive tests. These might be very valid measures to rate the behavioural effects of a drug, but the point is that when considering effect size it is crucial to bear in mind the nature of the assessments. It is also important to question whether there might be negative effects of taking a compound.

### The downside of cognitive enhancers

Like all drugs, those used with the aim of enhancing cognition can have side effects via body systems other than the brain. Thus, both AChEIs and methylphenidate frequently cause gastrointestinal upset or nausea, sometimes leading patients to discontinue medication altogether. These effects have the potential to offset any positive effects of the drug on overall performance, and also need to be borne in mind by anyone contemplating use of such drugs for non-medicinal purposes. More important from a cognitive neuroscience perspective is the ability of some drugs to impair certain aspects of cognition while simultaneously enhancing others in the same individual.

Thus, rivastigmine in healthy elderly subjects can improve learning on a motor task and making associations between symbols and digits, but can at the same time impair verbal and visual episodic memory [51]. Similarly, the dopamine agonist bromocriptine can enhance spatial WM while simultaneously impairing probabilistic reversal learning in young participants [52]. This finding echoes results in patients with PD: dopaminergic medication improves their performance on WM and task-set switching tasks, but degrades reversal learning [53,54]. It has been hypothesized that such opposing effects are due to ‘overdosing’ of ventral striatal areas involved in the latter, but replenishment of dopamine in dorsal striatal areas required for the former [53,55]. Thus, doses of dopaminergic medication sufficient to ameliorate motor function and some aspects of cognition in PD have the potential to worsen others.

Indeed, this conclusion might well be applicable to recent reports that some PD patients on dopaminergic agonists developed impulsive behaviours such as gambling, compulsive shopping and hypersexuality [56,57]. It has been reported that such behaviour in PD is often associated with the presence of dyskinesias, involuntary movements due to excessive dopaminergic stimulation [58], consistent with the notion that such impulse control disorders might indeed be associated with ‘overdosing’ of some basal ganglia regions. Importantly, reducing the dose of dopaminergic drugs often leads to reductions in impulsivity. These findings show that dopamine agonists in PD can have a spectrum of effects, both beneficial and harmful, on cognition and behaviour.

## Who benefits from cognitive enhancers?

A major theme that has emerged from studies of neurological patient groups is that there is a great variability of response, with many individuals not responding to treatment on (relatively crude) clinical measures, whereas others show a very strong improvement, for example in response to AChEIs [5,6]. Thus, although this group of patients demonstrates a modest average cognitive change overall, the effect is likely to be diluted by the fact that many individuals show very little benefit.

The same issue has arisen in investigations in healthy individuals: some subjects respond, whereas others might show little or no benefit. As we discuss below, recent investigations have begun to question whether such differences in outcome might depend on genotype and/or the baseline level of cognitive function. These considerations also raise concerns about what has become the standard method of performing clinical drug trials. Large-scale randomized controlled trials offer protection from false positive findings, but they also have the potential to discard the fact that some subgroups might benefit from a compound, whereas others might not.

What might be the cause of such variations in response? Several studies on the effects of dopaminergic drugs on WM in healthy volunteers support the conclusion that those who benefit most are low performers, such as those with low WM capacity or span. Thus, methylphenidate or dopamine receptor agonists such as bromocriptine improve WM updating or retrieval in people who were low performers on study entry, but can actually impair performance in participants with high baseline WM spans [47,59–62].

**Figure 3:**
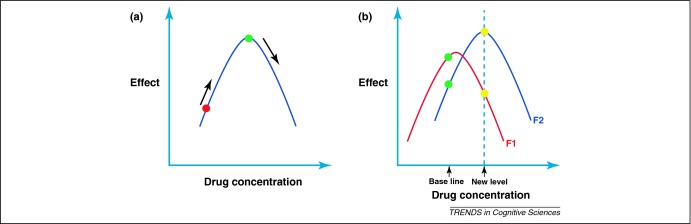
Drug concentrations modulate cognition across and within individuals. (**a**) Evidence from animal studies suggests that modulation of a cognitive process, such as spatial working memory, by a neurotransmitter such as dopamine might be described by an inverted U-shaped function. Too low or too high a concentration of dopamine in prefrontal cortex might not produce optimal functional effects. If an individual has low baseline concentrations of dopamine, small increases in concentration might help to improve performance (red circle). However, individuals with a higher baseline concentration of dopamine (green circle) might actually suffer an impairment of function on introduction of a drug. (**b**) Two cognitive processes within the same individual might have differential drug sensitivity (compare functions F1 and F2). In this case, this individual performed nearly optimally on cognitive function F1 but relatively poorly on function F2 (green circles) before drug administration. Administration of the drug led to an increase in neurotransmitter concentration from baseline levels. At the new drug level (dashed vertical line) performance on function 1 might theoretically decrease, whereas cognitive function 2 might now be optimized (yellow circles).

One possible explanation for such contradictory effects might reside in the classic inverted U-shaped relationship between cognitive performance and dopamine receptor (particularly D1 receptor) stimulation (Figure 3). Such effects have been known for a long time, with investigations in experimental animals revealing that both low and excessively high levels of D1 receptor stimulation in the prefrontal cortex can impair WM [63–65]. For optimal performance, a baseline level between these two extremes is required.

However, until recently, direct evidence in favour of this concept has been lacking in humans. New findings reveal that dopamine synthesis capacity in the caudate nucleus of the basal ganglia is lower in individuals with low WM spans compared to those with high spans [66]. Participants in this study were also investigated after taking bromocriptine or placebo. Ability to update reward predictions on a reversal learning task was improved by bromocriptine far more in individuals with low baseline dopamine synthesis capacity in the basal ganglia. Indeed, high-synthesis subjects were actually impaired in their performance [67].

More recently, it was demonstrated using radioligand positron emission tomography (PET) imaging that individuals with small levels of dopamine release induced by methylphenidate improved on a reversal learning task [31]. By contrast, participants with larger dopamine release in the caudate nucleus were impaired by the drug. Importantly, the authors also found that the most impulsive subjects (as indexed by their score on an impulsivity scale) were more likely to improve with methylphenidate. Thus, both baseline trait impulsivity and methylphenidate-induced dopamine release affected response to drugs.

The effects of methyphenidate on spatial WM in healthy subjects are also most prominent in individuals with the lowest performance [47]. In ADHD it has similarly been reported that children with the poorest sustained attention or highest baseline motor activity are most likely to respond to methylphenidate treatment [68]. The effects of baseline performance might also be evident for cholinergic modulation: whereas beneficial effects of donepezil on cognitive function were evident in healthy participants whose performance declined after sleep deprivation, those who were not much affected by sleep loss tended to deteriorate after donepezil intake [43,44]. Modafinil also seems to have the most prominent cognitive effects on attention and WM in subjects who have low baseline performance [69,70]. Interestingly, recent studies using magnetic resonance spectroscopy suggest that levels of GABA in specific brain regions predict differences in individual performance on cognitive tasks [71,72]. Thus, one reason for baseline performance modulation of response to drugs might be the baseline level of a neurotransmitter in a critical brain region or network.

### Effects of genotype on response to drugs

Genetic predictors of individual variability in response to treatments aimed at improving cognitive function would clearly be beneficial in effective targeting of therapeutic strategies. These effects might result directly from variations in efficiency of drug targets or indirectly via metabolic pathways or other risk genes. Several studies have suggested a role for polymorphisms in the catechol-O-methyltransferase (COMT) enzyme-coding region on chromosone 22 in WM [73]. COMT degrades catecholamines, including dopamine, at the synapse. Polymorphisms of the *COMT* gene seem to be associated with variability in human WM performance and associated brain activity, presumed to be via its putative influence on cortical dopamine levels [73].

Amphetamine responses might interact with COMT activity. When performing a test of cognitive flexibility – the Wisconsin Card Sorting Test – those with the higher-activity COMT Val-Val genotype improved, whereas those with the lower-activity Met-Met genotype deteriorated after a single dose of amphetamine. An inverted-U relationship between predicted cortical dopamine levels and performance is consistent with these findings (Figure 3).

Variations in COMT and the dopamine transporter gene (DAT) are both obvious candidates for modulation of response to psychomotor stimulant treatment in a condition such as ADHD. DAT is a major target of methylphenidate and amphetamine, and many treatments for ADHD, including the noradrenaline transporter inhibitor atomoxetine, are thought to increase cortical dopamine levels [74], consistent with a role for COMT. An association between good clinical response to methylphenidate and carriers of the high-activity Val polymorphism also suggests a role for cortical dopamine in mediating treatment response [75,76]. However, the influence of variable number of tandem repeats in the DAT gene on methylphenidate response seems to be mixed [77–79].

Apoliprotein E4 (*apoE4*), an allele of apolipoprotein E, which is involved in lipoprotein processing in cells, increases the risk of developing dementia later in life. Perhaps paradoxically, young healthy carriers of this genotype, who have a higher risk of cognitive decline later in life, actually show better performance on decision-making and prospective memory tasks compared to their *apoE3* counterparts [80]. Moreover, nicotine – but not dopaminergic drugs – potentiate the advantage in *apoE4* carriers, producing greater cognitive benefits in these individuals than in *apoE3* carriers on these tasks [80]. The reasons for this are unclear, but the findings suggest that some genetic variations influence the integrity of specific neurotransmitter systems, limiting the potential to improve function in response to drugs acting on the same systems.

For the AChEIs, extensive metabolizers of drugs as defined by gene variations in cytochrome P450 (a family of degradative enzymes) might show greater response to donepezil and rivastigmine [81,82]. This has been demonstrated using the Mini Mental State Examination (MMSE), which is a relatively crude bedside test of cognition; selective cognitive tasks have not been used to elucidate process-specific advantages.

## Drug effects and behavioural training

One area that is likely to develop in cognitive enhancement research is investigation of the interaction between drugs and behavioural approaches to improve cognition. There has been a great deal of recent interest in the potential for cognitive training, for example on WM tasks, to improve performance not only on these paradigms but also to generalize to other tasks in healthy people, as well as those with brain conditions such as ADHD [83,84]. fMRI studies in healthy participants have revealed alterations in activity across parietal and frontal regions during such training [85]. Intriguingly, radioligand PET imaging demonstrated associated changes in dopamine D1 receptor binding in parietal and frontal areas [86]. Thus it might be possible to visualize alterations in neurotransmitter systems as a function of cognitive training using brain imaging.

An important question for future studies will be whether there can be synergistic effects of behavioural training and cognitive-enhancing drugs. Such synergism has been demonstrated for learning of new material and levodopa in healthy subjects [87]. Whether such combined intervention might also be useful for cognitive deficits in brain disorders has yet to be explored in detail. However, there is emerging evidence of such effects. For example, both memantine and speech therapy improved dysphasia in stroke patients, but the combination of the two led to enhanced outcomes [88]. Demonstrations of network-level interactions for drug and cognitive training in this type of context would be an important way to investigate the mechanisms underlying such synergistic effects. Taking the effects of genotype, baseline cognitive performance and the nature of brain disorder in patients into account is likely to be an important factor in understanding such synergies.

## Concluding remarks

It would probably be fair to say that we are still in the first generation of studies to examine the potential for cognitive enhancement in humans. In both healthy individuals and many patient groups, the overall effects of drugs generally seem to be modest. However, there is evidence that there might be more significant effects in subgroups, such as those whose baseline performance is poorest or individuals with a particular genotype. Moreover, new drugs aimed at enhancing the phasic response of neurotransmitter systems, such as direct nicotinic agonists for the cholinergic system [34], might prove to have greater effects than existing modulators that globally increase levels of a neurotransmitter in a tonic fashion. The neurobiology underpinning the effects of cognitive enhancers and the mechanisms that determine responsiveness across individuals promise to be the focus of research in health and brain disorders in the future (Box 3).

## Figures and Tables

**Table 1:** Summary of the effects of some drugs frequently used as cognitive enhancers

Cognitive enhancer	Neuromodulatory mechanism	Cognitive functions improved	Known brain systems most affected	Currently recommended clinical use
Methylphenidate, amphetamine	Dopamine and noradrenaline reuptake inhibitors	Response inhibition, working memory, attention, vigilance	Frontoparietal attentional systems, striatum, default mode networks	ADHD, wake-promoting agent
Caffeine	Non-selective adenosine receptor antagonist	Vigilance, working memory, incidental learning	Frontal lobe attentional systems	–
Nicotine	Nicotinic cholinergic receptor agonist	Working memory, episodic memory, attention	Fronto-parietal attentional systems, medial temporal lobe, default mode networks	–
Modafinil	Unknown, but effects on dopamine, noradrenaline and orexin systems proposed	Working memory, episodic memory, attention	Frontal lobe attentional systems	Wake-promoting agent
Atomoxetine, reboxetine	Noradrenaline reuptake inhibitors	Response inhibition, working memory, attention	Frontoparietal attentional systems	ADHD, depression
Donepezil, galantamine, rivastigmine (AChEI)	Blocks enzymatic breakdown of acetylcholine	Episodic memory, attention	Frontal lobe attentional systems	Alzheimer's disease, PDD, DLB
Memantine	Noncompetitive, low-affinity, open channel blocker of the NMDA receptor	Episodic memory, attention	Frontal and parietal lobe	Alzheimer's disease

## Box 1 Measurement of cognitive enhancement in clinical trials

In clinical studies of neurodegenerative conditions – such as Alzheimer's disease, Parkinson's disease with dementia (PDD), dementia with Lewy bodies (DLB) and vascular dementia – the gold standard outcome measure has become the ADAS-Cog (Alzheimer's Disease Assessment Scale) [91]. This is a relatively short battery of cognitive tests covering memory, orientation, language, visual construction and limb praxis skills measured on a 70-point scale.

Drugs approved for use in these clinical conditions have demonstrated efficacy in changing this measure in the context of a randomized controlled trial (RCT), in which patients are randomly assigned either to drug or to placebo. Many trials have also revealed changes in CIBIC-plus (Clinician's Interview-Based Impression of Change) [92], ADAS-CGIC (Alzheimer's Disease Assessment Scale-Clinical Global Impression of Change) [92] or Neuropsychiatric Inventory (NPI) scores [93]. These scoring systems attempt to capture more global function or psychiatric effects of drug interventions.

For example, the CIBIC-plus is a semi-structured instrument that attempts to evaluate four areas: general, cognitive and behavioural functions and activities of daily living, based on the clinician's observations of the patient at interview, together with information supplied by a caregiver. By contrast, the NPI evaluates delusions, hallucinations, dysphoria, anxiety, agitation or aggression, euphoria, disinhibition, irritability or lability, apathy, aberrant motor activity, and night-time behaviour disturbances. It also relies on a structured interview with a caregiver who is familiar with the patient.

The problem with such scoring systems is that they are relatively crude and subjective. Many of them were developed for Alzheimer's disease and might not be as appropriate for other neurodegenerative conditions or for individuals performing in the normal range, but at risk of developing Alzheimer's disease. For example, fluctuations in attention or vigilance are a prominent feature of PDD and DLB whereas impairments in speed of information processing are common in vascular dementia. These aspects of cognition are not measured well by batteries such as ADAS-Cog.

Such scoring systems also often lack dynamic range and can be affected by ceiling or floor effects. Alternative measures comprising computerized batteries have therefore been used [36,94]. These can give more sensitive cognitive indices and reaction time measures can avoid saturation effects. However, they might be time-consuming to perform and require some degree of expertise to administer and interpret.

Similar issues also pertain to treatment studies of developmental disorders such as ADHD. Here, rating scales are also used as outcome measures, with trials showing relatively modest effects compared to placebo [90,95]. In ADHD too, experimental measures using reaction time indices, for example to assay response inhibition using the STOP signal reaction time task, might be more sensitive measures of the efficacy of drug interventions [96,97].

## Box 2 Neuroimaging of drug effects

Although a great deal of work has been performed on furthering our understanding of the actions of several cognitive enhancers at a cellular level, it is likely that this level of explanation will be insufficient, on its own, to account for the effects of drugs on cognitive performance in both healthy humans and those with brain disorders. Instead, more insight might be obtained from an understanding of the modulatory effects of drugs on large-scale brain networks underlying cognitive skills at the systems level. Early studies demonstrated how drugs such as AChEIs and methylphenidate might modulate visual attention and WM via effects on parietal, frontal and extrastriate occipital regions [47–49].

More recent investigations have focused on the effects of drugs on functional connectivity across a brain network. For example, reboxetine, a noradrenergic reuptake inhibitor, improved performance on a visuomotor task, an effect that was associated with enhanced effective connectivity between right hemisphere parietal and frontal regions, as well as their influences on left hemisphere regions [50]. Such approaches have also been used to examine more challenging effects, such as that of modafinil on the noradrenergic locus coeruleus, a very small nucleus located in the pons [98].

A different approach, applied to clinical populations, has been to examine brain metabolic network deficiencies associated with neurodegenerative conditions, such as motor and cognitive deficits in PD, using fluorodeoxyglucose PET [99]. Researchers have also started to use this methodology to investigate the effects of treatment at the network level, raising the possibility of producing a network-level account of how a drug might modulate function in a particular brain disorder. Importantly, different neurodegenerative diseases seem to have characteristically different effects on the resting-state functional connectivity across brain network nodes, as indexed by fMRI [100].

## Box 3 Questions for future research

• Can we improve outcome measures used to study the effects of drugs in clinical populations?
• Is it possible to improve prediction of which healthy individuals or patients might respond to a particular cognitive-enhancing drug?
• How do different neurotransmitter systems interact to modulate a particular cognitive function? Animal studies provide evidence of such interactions but there are few investigations in humans.
• Is there a role for combined treatments for neurological disorders, targeting different neurotransmitter systems?

## Glossary

Acetylcholinesterase: enzyme that breaks down acetylcholine at synapses.
Cholinergic system: nervous system pathways that use acetylcholine as a neurotransmitter. This includes cholinergic neurons in the basal forebrain that project to the cerebral cortex.
COMT (catechol-*O-*methyltransferase): enzyme that degrades catecholamines, including dopamine, at synapses.
DAT (dopamine active transporter): membrane-spanning protein that pumps dopamine from the synapse back into the cell, thereby reducing its synaptic concentration.
Dementia with Lewy bodies (DLB): form of dementia characterized by the presence of Lewy bodies (consisting of α-synuclein and ubiquitin proteins), closely related to Parkinson's disease with dementia (PDD).
Dopaminergic system: neurons that use dopamine as a neurotransmitter have cell bodies located in the midbrain. The mesolimbic pathway and mesocortical pathway originate in the ventral tegmental area to innervate the limbic system and cerebral cortex, respectively, whereas the nigrostriatal pathway projects from the substantia nigra to innervate the caudate and putamen.
Glutamate: ionized form of the amino acid glutamic acid; acts as an excitatory amino acid transmitter.
Heteroreceptors: receptors on axons that are specific for neurotransmitters released by other cells at axon–axon synapses.
Histaminergic system: neurons that use histamine as a neurotransmitter have cell bodies in the hypothalamus and project to brain regions including the cerebral cortex.
NMDA receptor: class of glutamate receptors activated by *N*-methyl*-*D*-*aspartate.
Noradrenergic system: neurons that use noradrenaline as a neurotransmitter project from cell bodies in the locus coeruleus in the pons to innervate the cerebral cortex.
Nucleus accumbens: part of the basal ganglia. Its inputs include dopaminergic neurons from the ventral tegmental area via the mesolimbic pathway.
Serotonergic system: neurons that use serotonin as a neurotransmitter project from cell bodies in the brainstem (notably in the raphe nucleus) to the cerebral cortex.
Working memory: process whereby information is held in mind for brief periods.
